# Outcomes to measure patient safety: the patient reporting and action for a safe environment (PRASE) trial

**DOI:** 10.1186/1745-6215-16-S2-P52

**Published:** 2015-11-16

**Authors:** Hannah Buckley, Kim Cocks, Rebecca Lawton, Jane O'Hara, Laura Sheard, Claire Marsh, Belen Corbacho Martin, Ian Watt, John Wright

**Affiliations:** 1 University of York, York, UK; 2 University of Leeds, Leeds, UK; 3 Bradford Institute for Health Research, Bradford, UK

## Introduction

The Patient Reporting and Action for a Safe Environment (PRASE) study, evaluated a ward-level intervention using ward-specific patient feedback to improve patient safety. We discuss choice of outcomes for patient safety trials.

## Trial design

A multi-centre, cluster randomised control trial was conducted over 12 months in 33 wards. Joint primary outcomes were a new routinely collected monthly ward-level measure, the patient safety thermometer (PST), and the patient measure of safety (PMOS) completed by around 25 study participants per ward at baseline, six and 12 months.

## Discussion of choice of outcomes

The PMOS had the advantage of providing a patient view on 44 safety related items; however researchers spent an average of 25 minutes with each patient collecting data and the measure was confounded as it also formed part of the intervention. Advantages of using routinely collected PST data included the objective nature of the data and low costs, however a ceiling effect was observed and spurious data could not be verified. The overall PST percentage of harm free care included previous harms and therefore could not be solely contributed to the trial intervention. We conducted a post-hoc analysis on the percentage of care free from new (ward-specific) harm.

## Conclusion

Given the use of ward-specific patient feedback, different areas were targeted for improvement on different wards meaning choice of outcome measure was difficult to identify. The PST allowed routinely collected data to be used as an outcome but lack of experience with the measure made it difficult to define a priori.

